# Improving In-Hospital Care For Older Adults: A Mixed Methods Study Protocol to Evaluate a System-Wide Sub-Acute Care Intervention in Canada

**DOI:** 10.5334/ijic.5953

**Published:** 2022-03-28

**Authors:** Malcolm B. Doupe, Jennifer E. Enns, Sara Kreindler, Thekla Brunkert, Dan Chateau, Paul Beaudin, Gayle Halas, Alan Katz, Tara Stewart

**Affiliations:** 1 Manitoba Centre for Health Policy, Department of Community Health Sciences, Rady Faculty of Health Sciences, University of Manitoba, Winnipeg, Canada; 2 George and Fay Yee Centre for Healthcare Innovation, Department of Community Health Sciences, Rady Faculty of Health Sciences, University of Manitoba, Winnipeg, Canada; 3 Rady Faculty of Health Sciences, University of Manitoba, Winnipeg, Canada

**Keywords:** sub-acute care, mixed methods, program evaluation, administrative data, implementation measures

## Abstract

**Introduction:**

Acute care hospitals often inadequately prepare older adults to transition back to the community. Interventions that seek to improve this transition process are usually evaluated using healthcare use outcomes (e.g., hospital re-visit rates) only, and do not gather provider and patient perspectives about strategies to better integrate care. This protocol describes how we will use complementary research approaches to evaluate an in-hospital sub-acute care (SAC) intervention, designed to better prepare and transition older adults home.

**Methods:**

In three sequential research phases, we will assess (1) SAC transition pathways and effectiveness using administrative data, (2) provider fidelity to SAC core practices using chart audits, and (3) SAC implementation outcomes (e.g., facilitators and barriers to success, strategies to better integrate care) using provider and patient interviews.

**Results:**

Findings from each phase will be combined to determine SAC effectiveness and efficiency; to assess intervention components and implementation processes that ‘work’ or require modification; and to identify provider and patient suggestions for improving care integration, both while patients are hospitalized and to some extent after they transition back home.

**Discussion:**

This protocol helps to establish a blueprint for comprehensively evaluating interventions conducted in complex care settings using complementary research approaches and data sources.

## Introduction

Older adults are the fastest growing segment of our population worldwide [1]. As life expectancy increases, so does the number of people who have complex functional challenges, chronic physical diseases, and mental illness [2,3,4,5,6]. Nationally and internationally, 8% of community-dwelling older adults 75-84 years old and 20% of those 85+ years old report challenges completing activities of daily living tasks such as walking unassisted or preparing meals [7,8]. Three-quarters of older adults are chronically ill [9] and one-quarter have three or more chronic diseases [7]. Compared to their healthy peers, older people with multi-morbidity are more likely to visit emergency departments (EDs) [10,11,12,13], and when hospitalized, they are at higher risk of experiencing infection [14], pressure ulcers [15], delirium [16,17,18] and medication-related errors [19,20]. A recent systematic review reports that nearly 20% of older people are re-admitted to hospital within 30 days of their index separation [21], further perpetuating this cycle of adverse events. The need for hospital re-admission is at least partially attributed to care integration challenges across providers and settings (e.g., poor communication, and inadequate discharge planning and implementation) [22,23,24,25,26]. Healthcare administrators, providers and researchers have for almost two decades been seeking to improve hospital-to-home transitional care for vulnerable older people [27,28,29].

According to Holland et al. (2003) [30], improving hospital-to-home transitional care requires strategies to enhance in-patient hospital care and discharge planning processes [31,32,33,34,35], and also to improve follow-up support once patients have relocated to other settings and/or care environments [36,37,38,39,40,41]. Multiple terms are used to describe the different components of this transitional care continuum (e.g., sub-acute care, intermediate care, post-acute care) [42], and in this protocol paper we describe an approach to evaluate a sub-acute care (SAC) program that was implemented in Winnipeg, Canada. SAC programs typically occur in units or facilities that are dedicated to provide time-limited care to medically stable (sub-acute) hospital inpatients, are generally designed to be integrated with and extend the traditional acute care model so that patients have shorter lengths of acute care hospital stay, involve multidisciplinary teams who collaborate to better prepare patients for discharge, and actively facilitate the patient transition process as part of hospital discharge [29,42,43]. It is important to note that (1) a range of SAC structures and processes exist, and that no single model has been shown to have clear and consistent advantages over others [29]; and (2) most of these interventions have been evaluated using metrics such as hospital length of stay [31,32,33], changes in patient function [31,32,34], and healthcare costs [31,35], collectively with mixed results. Recent reviews [21,27,44,45] and critiques [46] of this literature conclude that more diverse and synergistic evaluation approaches are needed to assess not only intervention effectiveness, but also to assess the ways in which transitional care interventions can be better integrated across providers and settings.

### Evaluation Frameworks and Study Goals

‘Transitional care’ is an umbrella term that describes how patients are transferred from one healthcare setting or care provider to another [24,42]. While several researchers have sought to enhance transitional care based on the seminal ideas of Coleman [28,47] and Naylor [39,48] (e.g., having multidisciplinary teams prepare and enact plans), ongoing improvements are required to better integrate care transitions from a health system perspective (e.g., improving coordination across sectors and providers) [49], from a ‘whole systems’ perspective (e.g., improving linkages between hospitals and communities) [50], and by more effectively engaging with providers (e.g., gaining their perspectives on how to reduce care fragmentation) [51] and patients (e.g., ensuring that strategies to improve transitional care meet their personalized needs) [52]. These perspectives have clear parallels to the evaluation framework proposed by Proctor et al. (2011) [53], who emphasize the importance of evaluating complex healthcare interventions using traditional Institute of Medicine [54] metrics (e.g., determining if transitional care interventions reduce hospital re-visit rates) combined with both implementation outcomes and client perspectives. Proctor et al. (2011) purport that implementation outcomes such as provider acceptability and perceived appropriateness (i.e., perceptions that a new approach has value over status quo and is compatible with provider beliefs and organizational culture), incremental cost and sustainability (e.g., opinions whether added time demands are worth it and can be sustained), and fidelity to the intervention (whether stakeholders can actually conduct core facets of the intervention as planned) are collectively essential to help differentiate between ineffective interventions and promising practices that have been poorly deployed (a termed call ‘implementation failure’ by Proctor et al. [2011] [53]). Similarly, Kreindler’s *Population, Capacity and Process* framework [55] proposes that effective system redesign requires a clear understanding and agreement about the intended target populations (e.g., if stakeholders feel that SAC patient eligibility criteria are clearly defined and correct), if the right kind and adequate number of providers is available to effectively care for these patients, and if processes have been developed to effectively link the two (e.g., properly matching provider care to patient need).

Guided by this knowledge, our study aims to evaluate an in-hospital SAC intervention implemented across the Winnipeg Health Region in the city of Winnipeg, Canada. We will evaluate the SAC intervention using a mixed-methods and sequentially phased research approach, first to evaluate intervention efficiency and effectiveness (e.g., use administrative data to report on the number and type of care transitions that SAC patients experienced preceding, during, and post discharge from SAC care), second to examine provider fidelity to the intervention (e.g., use chart audits to help define the type and frequency of care that SAC patients received, including the preparation of hospital-to-home discharge plans), third to investigate through provider interviews SAC implementation processes (e.g., how providers view the acceptability, perceived appropriateness, and implementation costs of SAC), and forth to determine through patient interviews additional perspectives about program success and/or the need for further modification. We will triangulate this complementary evidence to determine the extent to which and the reasons why the SAC intervention is succeeding or failing, and to understand the strategies required to better integrate this care transition program across providers and settings. Lessons learned from this research will help researchers and health system planners develop a roadmap for more comprehensively evaluating large-scale interventions conducted in complex healthcare settings.

### Local Context: The Health System in Winnipeg, Canada

Canada has a publicly-funded universal healthcare system that is governed by the Canada Health Act [56] and delivered provincially. Winnipeg is located in Manitoba, one of ten Canadian provinces with a population of 1.4 million people [57]. The province has five geographically diverse health regions; four of these regions are rural or remote and the Winnipeg Health Region is the only large metropolitan area (population 817,000). Most tertiary care specialized services in Manitoba (e.g., cardiac surgery, neurology, and intensive care) are provided in Winnipeg through six hospitals comprising 2,085 inpatient beds.

In recent years, Winnipeg has had one of the longest ED wait-times in Canada [58], above average lengths of inpatient stay [59], and large numbers of alternate level of care hospital patients [60]. Three recent reviews have also stated that Manitoba’s health system is both fragmented and inefficient [61,62,63]. In 2017, the Winnipeg Health Region responded by launching a major system transformation (called *Healing our Health System*) designed to consolidate clinical services and staff resources [64]. While three Winnipeg Health Region hospitals (1,506 beds) have remained as traditional acute care facilities, the remaining three hospitals (579 beds) were re-purposed to provide sub-acute care, specialized services (e.g., orthopaedic surgery, dialysis, geriatric care), and to a lesser extent transitional care to nursing homes. EDs at the three sub-acute sites were converted to urgent care departments, intensive care units were consolidated to the three acute hospital sites, and revised EMS-ambulance routing algorithms and public media campaigns were developed to help stream patients to the appropriate site based on acuity. As well, a ‘Home is Best’ policy was created, from which enhanced hospital-to-home care transition services (Priority Home [65] and Rapid Response Nursing Teams [66] were developed.

### The In-Hospital Sub-Acute Care Intervention

Winnipeg implemented SAC in a phased manner; 89 acute care beds in one hospital were converted to SAC in October 2017, followed by 222 beds from two additional hospitals in June/July 2019. As part of the Winnipeg Health Region transformation strategy, SAC was developed to help improve the efficiency of the overall hospital system, first by concentrating resources for higher acuity patients at a smaller number of sites, and second by allowing SAC patients to be managed in units with lower physician and nursing ratios but with more allied health staff. Potential benefits of SAC are therefore at both the system level (e.g., more efficient care for both high and lower acuity patients) and the patient level (e.g., enabling SAC patients to receive more patient-centred interprofessional care, and better preparing them to return home).

Sub-acute care is a subservice of the Winnipeg Health Region general medicine program. Similar to the criteria used by others [31,33], patients are eligible for SAC if they (a) still require general but not acute levels of medical care, (b) have stable vital signs and no oxygen requirements, (c) are unlikely to decompensate medically, and (d) do not require ongoing special care (e.g., rehydration) or significant behaviour therapy. While SAC patients are admitted via three major pathways (on-site urgent care, off-site EDs, and off-site acute care units), ambulance routing procedures and public information campaigns have helped to ensure that most patients with sub-acute care needs are admitted from on-site urgent care departments. A Central Bed Access service monitors system-wide capacity and flow and helps to prioritize and coordinate off-site transfers into SAC.

To support SAC implementation, interprofessional teams were redeployed from existing hospital units; these staff received additional training in dementia and gerontological care, and in SAC best practices and care plan procedures. SAC care is initiated by registered nurses and is authorized/adjusted by the attending family physician within one day of patient admission. Interprofessional teams participate in daily rounds to create and amend care plans as needed, and to discuss barriers to patient progress and transition. All aspects of patient care (e.g., team meeting dates, decisions made) are documented using clinical decision software. Patient transition is a team-consensus decision based on medical (e.g., stable vitals) and additional patient (e.g., functional status) criteria. Additional details about the SAC intervention are provided in ***Table 1***.

**Table 1:** Sub-Acute Care* Intervention Components.


COMPONENT	DESCRIPTION

Service Purpose	To (1) provide quality care to patients who require daily individual assessment, general medical care, and interventions to enhance their functional, cognitive, psychosocial, and spiritual well-being; and (2) liaise with various community-based and institutional programs to facilitate out-of-hospital patient transitions.

Patient Profile	Adult patients 18+ years old with a general medical diagnosis and who have stable vitals; low/stable oxygen requirements; are unlikely to decompensate, and; do not require acute specialised hospitalized services. The target length of stay for SAC patients is 14–16 days.

Admission Pathways & Processes	SAC patients are admitted (1) directly from the onsite urgent care departments (primary pathway); (2) offsite from one of three emergency departments, or; (3) via transfer from an acute care medicine bed. A Central Bed Access service provides a gate keeping function, usually prioritizing direct onsite (urgent care) admissions.

Team Composition, Recruitment & Training	SAC patients are visited by an attending physician at least once daily and nursing care is provided by a mix of registered and licensed practical nurses. SAC teams are comprised of an extensive complement of allied health disciplines including clinical nutrition, speech-language pathology, occupational therapy, physiotherapy, pharmacy, respiratory therapy, social work, spiritual health and therapeutic recreation. Specialist consults from off-site programs such as psychiatry, geriatrics, and orthopedics are available as-needed. Hospital-based nurse case coordinators liaise with a range of community and institutional (e.g., nursing home) staff to facilitate out-of-hospital care transitions. SAC staff were redeployed from previously-undifferentiated (acute/sub-acute mix) hospital units. Teams received specialized training in dementia care and in use of the National Early Warning System (identifies patients at risk of clinical deterioration). Teams also received a patient flow guide, and an operations manual that defines roles, accountability, best practice procedures, and tools to help support & evaluate patient progress.

Care Planning & Communication	Care plans are initiated by the attending family physician within one day of patient admission. Four processes are used to support care planning, delivery & inter-professional collaboration. These include: **1)**Daily Action Rounds: The entire care team meets daily to review care plans & to address barriers to patient discharge. **2)**Complex Case Rounds: Teams have dedicated time to develop care plans (e.g., engaging with off-site staff) for particularly complex patients. **3)**Bedside White Boards: These tools are used to communicate important information to the patient/family about care goals, to provide an estimated discharge date, and to name the care team members. **4)**Patient Flow & Clinical Decision Software: All staff have access to real-time data on wait times, patient admissions, discharges and bed availability. Care plans, staff meeting dates, patient progress and barriers to discharge are updated continuously using clinical decision software.

Discharge Process & Criteria	Discharge planning begins at the time of patient admission and is supported by clinical decision software. Patients are eligible for discharge when: **1)** Vital signs are stable, nausea/vomiting is controlled, pain is appropriately managed, oxygen saturation is above 90%, lab values are in an acceptable range, and patients are able to void sufficiently and independently (with or without support), AND; **2)** Team members agree that the patient is ready for discharge from a functional, psychosocial and cognitive perspective.


* Termed “Lower Acuity Care” in the Winnipeg Health Region.

## Methods

### Research Questions

Guided by the aforementioned frameworks proposed by both Proctor et al. (2011) [53] and Kreindler (2017) [55], we have developed six research questions to evaluate the SAC intervention in Winnipeg:

1. Who is the population of SAC patients and did this change with time (from early to later stages of the intervention)?
2. Does SAC operate efficiently, e.g., how many and what type of transitions did SAC patients experience, what were their hospital lengths of stay, what type and intensity of care (priority home and rapid response nursing home care services, follow-up visits with primary care physicians) did SAC patients get following hospital discharge?
3. How effective is SAC, e.g., did it result in prolonged community living, fewer emergency department visits and lower hospital re-admission rates?
4. To what extent did providers deliver the types of care intended for patients while in a SAC bed, and did this vary by select patient group?
5. Do providers feel that SAC is a valued and suitable intervention to enhance hospital care? What factors facilitated and/or impeded SAC implementation, and how can SAC be better integrated across providers and settings?
6. What do patients and their family/friend caregivers think of SAC and what are important measures of success and failure from their perspective? Which aspects of SAC do they feel worked well, and what recommendations do they have for change?

### Overall Evaluation Strategy

An overview of our evaluation strategy is provided in ***Table 2***. We will use an explanatory mixed methods sequential design, in part so that qualitative findings will help to explain, elaborate and contextualize quantitative results [67]. In Phase 1 (months 1–12), administrative healthcare use records will be linked, first to define the profile of SAC patients and to assess whether these patient characteristics changed during the intervention, and second to evaluate SAC using measures of efficiency and effectiveness. The latter analyses will be conducted overall and across time periods (earlier versus later stages of SAC) and patient subgroups. These findings will be used in part to guide audits of SAC patient medical charts in Phase 2 (months 12–15), designed to assess provider fidelity to SAC standard operating procedures. Qualitative interviews will be conducted in Phase 3 (months 16–24), designed to explore the experiences that providers and patients (as well as their family/friend carers, where possible) have had with SAC, and to identify their recommendations for change.

**Table 2:** Overview of Evaluation Domains, Data Sources and Study Timeline.


PHASE	EVALUATION DOMAIN	DATA SOURCE	TIMELINE

**Phase 1**	Healthcare Service Outcomes **-** efficiency, effectiveness of the intervention	Linked person-level administrative healthcare use records	Months 1–12

**Phase 2**	Implementation Outcomes **-** fidelity of the provider to the intervention	Medical chart audits	Months 12–16

**Phase 3**	Implementation Outcomes **-** acceptability and feasibility of the intervention **-** barriers and facilitators, strategies to enhance integrated care	Provider interviews	Months 16–24

	Patient/Informal Caregiver Experiences **-** measures of success and failure of the intervention, strategies to enhance integrated care	Patient/informal caregiver interviews	

**Phase 4**	Integration of the Results	Phases 1–3	Months 24–36


## Results

### Evaluation Phase 1 – Linked Administrative Healthcare Use Records

Data Sources and Study Variables. Manitoba’s population-based healthcare system data repository is housed at the Manitoba Centre for Health Policy. The Repository contains information on every registered Manitoban (>99% of the population) since 1970. While Repository data are de-identified (names and addresses removed), files are linked using a scrambled 9-digit personal health identification number attached to each record using a secure standard process [68].

Key repository files to be used in this study are listed in ***Table 3***. These data were selected given our teams’ experience measuring care transitions [69,70,71], to reflect the ‘whole systems’ perspective of care transitions (e.g., ensuring that we describe key healthcare transitions leading into, during, and post hospital care), and to reflect the major outcomes assessed in the academic literature [21,72]. These data will be used for two purposes. First, the Admission, Discharge and Transfer file provides date-stamped and bed-specific durations of hospital stay, from which we can define the SAC cohort. These patients will be characterized by (a) socio-demographic factors including patient age, sex, marital status and income quintile; (b) the presence of chronic diseases (e.g., arthritis, COPD, diabetes, ischemic heart disease, stroke, Alzheimer’s disease/dementia, and a measure of multi-morbidity) using validated algorithms [73] based on past hospitalizations, ambulatory care physician visits, and in some instances, prescription drug dispensations; and (c) their index hospitalization (i.e., hospital patient service [74] and procedural codes [75] will be used to help define patients’ acuity status during this hospital stay).

**Table 3:** Administrative Datasets from the Data Repository used in this Study.


REPOSITORY FILE	PURPOSE

Population Repository	This file defines registered Manitobans by key socio-demographic factors (age, sex, marital status, income quintile) and death date (using the Repository cancellation code).

Admission, Discharge, and Transfer File	This file provides date-stamped and bed-level hospital use data and will be used to (1) identify (using bed identifiers) SAC patients, and (2) define detailed hospital transitions pathways leading to and from SAC units.

Hospital Discharge Abstract Database	This file provides date- and site-stamped data on hospital use parameters, and up to 26 international classification of disease (ICD-10-CA) codes to define patient’s admitting diagnosis and complications that arise after hospital admission.

Emergency Department Information System	This file provides date- and time-stamped records of emergency department visits by site and patient acuity.

Medical Claims	This file provides date-stamped record on ambulatory care physician visits. One ICD-9-CM (clinical modification) code is provided per visit.

Home Care	This file provides the start- and end-date, volume and type of home care services received by each registered Manitoban (e.g., to identify prevalence [before SAC] and incidence [after SAC] home care users). Use of the Priority Home and Rapid Response Nursing programs are included in this overall file.

Nursing Home	This file provides the admission and exit date of nursing home use (to determine SAC disposition status).

Supported Living	This file provides the admission and exit date of congregate community housing use (to determine SAC dispositions status).

Drug Program Information Network	This file provides dispensation-level data on prescription drugs dispensed from retail (not in hospital) pharmacies (i.e., by their anatomical, therapeutic & chemical classification system).


Administrative healthcare use records will also be used to examine the following healthcare use outcomes as discussed by Proctor (2010) [53] and in keeping with the Institute of Medicine [54]:

- Efficiency. This includes transition pathways into SAC (e.g., directly from home or after multiple ED visits); intra-hospital transition patterns (e.g., one versus multiple transitions preceding SAC, repetitive bouts of SAC and non-SAC care, the proportion of total hospital time spent in SAC); and continued support post hospital discharge (e.g., how many patients received home care post hospital discharge, the length of time between discharge and their first home care visit; and the type, intensity, and duration of home care services that they received).
- Effectiveness. We will define SAC patients’ post discharge rates of ED visits and hospital re-admissions; and time prior to death and nursing home admission. As per the literature [21,72], these outcomes will be measured at 30, 90 and 180 days after hospital separation.

Cohort Development & Quantitative Analysis Plan. We will allow for a three-month ‘settling in’ period during which healthcare use data will not be used in the study. While SAC commenced in October 2017, healthcare use data will be analyzed from January 2018 to April 2020 (26 months; the study period).

Using the methods defined by Schneeweiss et al. (2009) [76], we will use a machine learning algorithm to generate a propensity score of SAC patients by their existing chronic disease, previous healthcare use, and index hospital visit profile. This propensity score will be used to match truly exposed (SAC) patients to a cohort of unexposed patients with similar profiles (hereafter referred to as ‘SAC look-alikes’). This approach has been shown to produce results similar to RCTs by identifying maximally important confounders [77,78]. A random sample of conventional acute care patients (i.e., those who had a negligible likelihood of being exposed) will also be developed based on this knowledge. These approaches will also be used to define a pre-SAC (April 1, 2015 to September 30, 2017) comparison group for the combined SAC and SAC look-alike patients, and a separate pre-SAC group for the conventional acute care patients. In keeping with the overall goals of SAC (e.g., to enhance quality of care for both SAC *and* acute care patients), we hypothesize that (pre-SAC to SAC) improvements in effectiveness and efficiency will be significantly greater for each of the SAC and conventional acute care patient groups versus the SAC look-alike group.

Three complementary stages of quantitative analyses will occur. First, as per Kreindler’s *Population, Capacity and Process* framework [55], descriptive and multivariable modelling will occur to define the main sociodemographic and chronic disease profile of SAC patients, to compare this profile to the SAC look-alike and conventional study groups, and to assess if the predominant profile of SAC patients changed during the course of the study period. Second, process control charts [79] will be used to determine the pattern of healthcare use outcomes during the study intervention (e.g., if outcomes improved suddenly and dramatically versus slowly over time), overall and by the major sub-groups of SAC patients. This strategy will use historical (pre-SAC) data to create ‘usual care’ sigma values, and 26 data points (duration of SAC in months) to apply the Western Electric Rules for detecting non-random deviations from this historical data [80]. Third, these findings will be used to guide multivariable statistical modelling to determine study group-specific improvements in healthcare use pre versus during the SAC intervention, and whether the numerical size of these improvements varied by key predetermined factors (e.g., by major patient groups, from early to later stages of the intervention). Healthcare use outcomes will follow either a binomial distribution (e.g., hospital re-admission) in which case we will use logistic regression, or a count distribution (e.g., physician & ED visits) in which case we will use a Poisson or negative binomial regression. Because there are a small finite number of hospitals (N = 3) where the SAC program operates, multilevel modelling techniques will be used to account for data clustering.

Sample Size Estimation. From discussions with planners, we anticipate having data on ~2,340 SAC patients during the study period (on average, three patients are admitted daily into SAC). Sample size estimates were calculated using 90-day hospital re-admission rates as the outcome. Using data from the systematic review of Le Berre et al. (2017) [21], we anticipate secular re-admission rates to range between 25% and 35%, and about an 18% reduction in re-admission rates associated with SAC. Based on these estimates, we will require at least 1,000 patients to detect statistically significant events. Given this sample size calculation relative to our projected cohort size, we anticipate conducting all phase 1 data analyses across hospital sites combined. Descriptive results will first be compared across hospitals, to justify this decision. Sample size calculations are provided in **Additional File 1**.

### Evaluation Phase 2 – Chart Audits

Patient Selection. SAC care strategies are documented in a clinical decision software program (see ***Table 1***). Following the 10% rule of thumb proposed by Gregory et al. (2008) [81], these data will be analyzed for ~200 SAC patients selected randomly within pre-defined strata defined by Phase 1 results (e.g., by dementia status if Phase 1 results show that SAC effectiveness differs significantly by this versus other patient groups), thus permitting sub-group comparisons. Our selection of patient strata will be made by team consensus after reviewing phase 1 results, and with consideration of the existing academic literature (e.g., Le Berre et al. [2017] conclude that transitional care interventions may be less effective for people with congestive heart failure [21]). Combining Phase 1 and 2 results in this way, along with the interviews in Phase 3, will help us to understand the extent to which SAC ‘works’ overall and for sub-groups of patients (i.e., the concept of intervention failure as defined by Proctor et al. [2011] [53]), or conversely, whether provider fidelity to core SAC practices varies across patient groups (the concept of implementation failure as defined by Proctor et al. [2011]).

Data Collection. SAC requires providers to undertake specific activities within defined time limits (e.g., conduct a full patient assessment within 24 hours, immediately establish a rehab plan, conduct daily team meetings, provide regular rehab therapy; see ***Table 1***). Clinical decision software will be reviewed, and for each criterion teams will receive a score of ‘no compliance’ (i.e., care activities did not occur), ‘partial compliance’ (i.e., activities were documented but occurred less frequently than required), and ‘full compliance’ (frequency of activities documented as per guidelines). We will present results descriptively and explore any associations between patient characteristics and provider compliance.

Analysis Plan. A draft of the audit tool is provided in **Additional File 2**. To refine this tool, team members will first conduct an informal focus group with 4-6 providers, asking how care is recorded and the meaning of the terminology used. Two auditors will then independently review the data for 15 patients, compare results and adjust the audit tool as necessary. Additional strategies will be used to optimize rigor. First, auditors will not be shown the administrative data results and will also be blinded to the different strata (e.g., patients with and without dementia) of charts selected. Second, auditors will review ~20 of the same charts early in data collection to measure inter-rater reliability. Re-training will occur if kappa values [82] from this comparison are below 0.6.

### Evaluation Phase 3 – Healthcare Provider Interviews

We will interview 4-6 key informants (planners and providers involved in the program) to verify that we properly understand how the SAC intervention should ideally work, eligibility criteria for SAC, and how these processes have changed with time. These key informants will also help us to refine provider interview questions and to identify important groups of providers to interview.

Interviews will then be conducted with providers who refer patients to SAC units and also with providers comprising the interprofessional SAC care team (see ***Table 1***). Using purposive sampling to ensure representation of both referring and receiving staff, participants will be recruited via e-mail, word of mouth and staff meetings, and through snowball sampling. Sampling will proceed until data have reached thematic saturation. Based on the experiences of others [83], to reach saturation we anticipate conducting interviews on about 20 SAC-referrers and 20 SAC-providers.

Data Collection & Analysis Plan. Interviews will be conducted using a semi-structured guide. Interview questions will inquire about the SAC population (e.g., if respondents feel that the ‘right’ patients are currently being served by SAC, and if not, who should/should not be accepted), provider knowledge and awareness (e.g., about the overall SAC purpose and individual responsibilities), the SAC intervention (e.g., if people feel that SAC is an acceptable and feasible alternative to status quo, suggestions for improvement), implementation facilitators and barriers (e.g., whether clear duties and operational guidelines are defined; if staff feel they were adequately trained, are sufficient in number, and as a team have the right complement of expertise to care for SAC patients), and strategies to enhance integrated care (e.g., between referring and receiving staff, amongst the SAC multidisciplinary team, between the hospital and community sectors). Additional interview questions will be formulated depending on the results of Phase 1 (e.g., asking providers to help explain why SAC ‘worked better’ for some patient groups than others, to provide potential solutions to demonstrated challenges).

Provider interviews will be audiotaped and transcribed verbatim. Qualitative analyses, proceeding from a interpretivist paradigm, will focus on describing the semantic content of participants’ responses. The analysis will have deductive and inductive phases. First, content analysis, guided by a preliminary coding scheme informed by Proctor et al. (2011) [53] and Kreindler (2017) [55] will provide a descriptive account of participants’ responses. The data will then be revisited to identify new themes inductively, paying special attention to previously uncoded text. Differences in themes across participant groups (e.g., referrers vs receivers) will be explored to determine the extent to which understandings are shared or divergent. Each analysis phase will be undertaken by two independent coders, who will meet frequently to discuss discrepancies and reach consensus. Preliminary findings will be shared with our diverse stakeholder team to enable participant validation of themes and key findings.

### Evaluation Phase 3 – Patient & Caregiver Interviews

Selection of Interviewees and Data Collection. Team members will randomly select about 30 charts of SAC patients discharged from hospital in the last six months. While this timeline will not permit us to compare the perspectives of patients who received care in the earlier versus latter stages of SAC, it was chosen to help minimize recall bias. To help minimize selection bias, we will attempt to recruit patient participants in proportion to SAC cohort by age group (<65, 65–84, 85+ years old) and sex (about five participants per age- and sex-category) and more generally by socio-economic status (e.g., lower versus higher income quintiles) and hospital (three sites). Patient names will be sent to the Manitoba Government, who will in turn send patients an information letter inviting them (and by extension, their family/friend caregivers) to contact researchers directly for more information and an interview. Patients within each stratum will be oversampled by 50% to account for non-responds and those who do not consent to participate in the study. Interviewers and analysists will also be blinded to select strata criteria (e.g., hospital status) to help further minimize bias. Lastly, at the beginning of each interview, participants will be asked to recall some basic details about their SAC care (e.g., the approximate dates that they were hospitalized and their duration of stay, the type of services they received upon returning to the community). These participant responses will be compared to our phase 1 records, and if similar, we will conclude that participants have sufficient recall to participate in the interview. People who cannot accurately recall these basic details (e.g., that they were hospitalized or received home care post return the community), will not be eligible to complete the interview.

Eligible interview participants, and where possible, their family/friend carers, will be jointly asked about their awareness of SAC (e.g., how they were introduced to the program), their perceived effectiveness of the intervention, and important outcomes and suggestions they have for making improvements. A more detailed interview guide will be developed in consultation with our patient partners, who will in turn gain feedback from a larger patient and caregiver advisory group. Patient and caregiver interviews will be audiotaped and transcribed verbatim. Deductive and inductive analysis will be carried out in the same manner as described above.

### Evaluation Phase 4 – Integrating Findings Across Research Phases

Quantitative and qualitative methods will be integrated at several junctures. First, as research phases are sequential, findings from earlier phases will guide the sampling frame and questions posed in latter phases. Second, towards the end of provider interviews in Phase 3, we will give respondents the opportunity to react to specific Phase 1 and 2 findings, inviting them to offer potential explanations of these results. Third, preliminary quantitative and qualitative findings will be combined into a matrix to help identify convergent and divergent results. Following the strategies proposed by Rossman and Wilson (1985)[84], the quantitative and qualitative findings will be synthesized to leverage analytical corroboration and elaboration, which will be discussed among the team to help initiate new or modified interpretations of existing study results, and/or to suggest potential areas of follow-up analysis.

## Discussion

This research uses a mixed method design to evaluate a sub-acute care intervention that has been implemented across the Winnipeg Health Region in Manitoba, Canada, designed to improve in-hospital and hospital-to-home care transitions for older adults. Care transitions involve multiple players (e.g., patients, providers, decision makers), cultures and settings, and are often complicated by suboptimal communication and coordination processes that create bottlenecks and result in fragmented and unsafe care [85,86]. More patient-centred and integrated care approaches are needed to help ensure that patients receive a seamless continuum of services that respond to their changing needs as they transition from hospital to home. The literature on care transition interventions highlights a number of challenges related to the outcomes measured, limited descriptions of implementation processes, and a lack of knowledge about the ways in which care setting contexts influence intervention success [27]. This protocol describes a mixed methods research approach that aims to mitigate some of these challenges by triangulating the knowledge generated from complementary sources. Administrative healthcare use records, medical chart review data, and provider and patient/caregiver interviews will be used to describe the intervention successes and challenges from different perspectives, to identify intervention components that work and that require change, and to examine implementation processes (e.g., additional training) required to optimize integrated care approaches. The lessons learned from this research will help healthcare planners to further adapt the SAC intervention, and will help to produce a methodological roadmap for evaluating large-scale interventions conducted in complex healthcare settings.

### Integrated Knowledge Translation Approach

Our research team consists of seven multidisciplinary health services researchers; five decision makers representing the Manitoba Government, Winnipeg Health Region and Shared Health; care providers; and two patient representatives. Guided by the Canadian Institutes for Health Research’s integrated knowledge translation approach, this team will meet at the beginning, mid-point, and end of each study phase to interpret and contextualize findings, and to discuss how the new knowledge should be used to help refine subsequent research questions and methods. We have created an Operations Committee consisting of SAC program planners and providers, to help ensure that the detailed research questions posed in each study phase are appropriately contextualized and hold meaning to program providers. As well, one of our team members (GH) will work with community advisors to help ensure that we effectively engage with these individuals. We have also allotted funds to pursue various non-traditional knowledge translation activities including creating an infographic, writing op-eds and media releases, and hosting a public discussion forum to reach a diverse audience. An end-of-grant workshop will be held with key decision makers from across Western Canada to share learnings and to facilitate future research (e.g., prospective evaluation) endeavours.

### Strengths & Limitations

The major strengths of this research include the rich quantitative and qualitative data sources combined with our synergistic analysis plan, and our integrated knowledge translation activities involving local and national partners. While Phases 1 and 2 of the research will assess, in part, post-hospital discharge processes (i.e., in Phase 1 we will determine the proportion of SAC patients who received home care post hospital discharge; in Phase 2 we will assess the extent to which providers pre-emptively made these transitional care plans), due to budget constraints (a) provider interviews will be confided to those giving in-hospital care; and (b) we have limited ourselves to conducting 30 patient interviews, which is a small in comparisons in the total number of patients that we expect in our cohort (~2340). During these patient interviews, we will ask respondents to provide suggestions to improve both in-hospital and hospital-to-home transitions.

## Conclusion

The proposed research will comprehensively evaluate an intervention designed to improve hospital-to-home care transitions for older adults. The lessons learned from our evaluation approach have application for evaluating additional interventions conducted in complex healthcare settings.

## Additional Files

The additional files for this article can be found as follows:

10.5334/ijic.5953.s1 Additional File 1. Sample Size Calculation.

10.5334/ijic.5953.s2 Additional File 2. Draft Capture Sheet for Subacute Care Chart Audit.

## Abbreviations

SAC: sub-acute care; ED: emergency department; EMS: emergency medical services; ICD: International Classification of Disease; HIPC: Health Information Privacy Committee.
