# Supplementary material for: Improving In-Hospital Care For Older Adults: A Mixed Methods Study Protocol to Evaluate a System-Wide Sub-Acute Care Intervention in Canada
Source: Int J Integr Care. 2022 Mar 28;22(1):25. doi: 10.5334/ijic.5953 (PMC8973798; doi:10.5334/ijic.5953)
Supplement: Additional File 1. — Sample Size Calculation. [file ijic-22-1-5953-s1.pdf]

Additional File 1: Sample Size Calculation

Sample Size Calculations Derived from Figure 3B of Berre et al*

(Comparing 90-day Hospital Re-admission Rates between Transitional Care & Usual Care Study Groups)

	Data Extracted from Table				Calculations from Table		
	Transitional Care		Usual Care (UC)		Rate (TMT group)	Rate (UC)	Rate Ratio (TMT/UC)
	Events	Total	Events	Total			
Al-Rashed 2002	8	43	28	40	0.19	0.70	0.27
Bowles 2011	15	101	20	116	0.15	0.17	0.86
Chow 2014	62	183	59	98	0.34	0.60	0.56
Coleman 2006	63	379	84	371	0.17	0.23	0.73
Duffy 2010	2	15	3	17	0.13	0.18	0.76
Harrison 2002	21	92	31	100	0.23	0.31	0.74
Hermiz 2002	16	67	14	80	0.24	0.18	1.36
Jaarsma 1999	22	84	29	95	0.26	0.31	0.86
Laramee 2003	49	131	46	125	0.37	0.37	1.02
Ledwidge 2003	2	51	12	47	0.04	0.26	0.15
Lopez 2006	12	70	24	64	0.17	0.38	0.46
Naunton 2003	16	57	29	64	0.28	0.45	0.62
Nazareth 2001	164	171	176	176	0.96	1.00	0.96
Pekmezaris 2012	42	83	25	85	0.51	0.29	1.72
Rich 1995	41	142	59	140	0.29	0.42	0.69
Riegel 2002	44	130	94	228	0.34	0.41	0.82
Riegel 2006	26	69	26	65	0.38	0.40	0.94
Stromberg 2003	16	52	17	54	0.31	0.31	0.98
**Average**					0.30	0.39	0.81
**25th percentile**					0.18	0.27	0.64
**median**					0.27	0.34	0.79
**75th percentile**					0.34	0.42	0.95

Sample Size Calculations Pending Expected Rate Ratios and Four Potential Usual Care (UC) Re-admission Rates. Assumed Power = 0.80

**Estimated N**	**Rate Ratios Stratified by Usual Care Re-admission Rates** 0.2	0.25	0.3	0.35
400	0.70	0.73	0.75	0.77
600	0.75	0.77	0.79	0.80
800	0.78	0.80	0.82	0.83
1000	0.80	0.82	0.83	0.85
1200	0.82	0.83	0.85	0.86
1400	0.83	0.85	0.86	0.87
1600	0.84	0.85	0.87	0.88
1800	0.85	0.86	0.87	0.88
2000	0.85	0.87	0.88	0.89

Notes:

1) While 30-day re-admission rates are not available for frail older Manitobans, Cui et al. (2015)** report that 48.1% of Winnipeg people age 75+ years old are re-admitted to hospital within one year of separation.

2) Shaded areas in this table reflect the average or median rate ratios reported in the literature, by various usual care (UC) 30-day re-admission rates. Assuming that SAC will

* Berre M Le, Maimon G, Sourial N, Guériton M, Vedel I. Impact of transitional care services for chronically ill older patients: A systematic evidence review. *J Am Geriatr Soc*. 2017;65(7):1597-1608. doi:10.1111/jgs.14828

** Yang Cui, Mahmoud Torabi, Evelyn L Forget, Colleen Metge, Xibiao Ye, Michael Moffatt, Luis Oppenheimer. Geographical variation analysis of all-cause hospital readmission cases in Winnipeg, Canada. BMC Health Services Research (2015) 15:129
