# Supplementary material for: Improving In-Hospital Care For Older Adults: A Mixed Methods Study Protocol to Evaluate a System-Wide Sub-Acute Care Intervention in Canada
Source: Int J Integr Care. 2022 Mar 28;22(1):25. doi: 10.5334/ijic.5953 (PMC8973798; doi:10.5334/ijic.5953)
Supplement: Additional File 2. — Draft Capture Sheet for Subacute Care Chart Audit. [file ijic-22-1-5953-s2.pdf]

Additional File 2: Draft Capture Sheet for Subacute Care Chart Audit

Unique Study ID:____________

Auditor Initials:______________

Today's Date of Review Date (Year/Month/day)________________Time (24-hour)____________

1. Patient Gender: ☐ **Male** ☐ **Female**

2. Patient Date of Birth: (YYYY/Month/day) ________________

3. **Date of Admission to Sub-Acute Care** (YYYY/Month/day)______Time (24-hour)____

4. Patient Needs Reported upon Admission to SAC

a) First Assessment Date (Month/day)__ Time (24-hour)___________

b) Evidence that patient's needs were assessed in the following domains

Medical	☐ No	☐ Yes	Date (Month/day)__________	Time (24-hour)______
Functional	☐ No	☐ Yes	Date (Month/day)__________	Time (24-hour)______
Cognitive	☐ No	☐ Yes	Date (Month/day)__________	Time (24-hour)______
Mental	☐ No	☐ Yes	Date (Month/day)__________	Time (24-hour)______
Social Support	☐ No	☐ Yes	Date (Month/day)__________	Time (24-hour)______
Prescription Drug	☐ No	☐ Yes	Date (Month/day)__________	Time (24-hour)______
Other _____	☐ No	☐ Yes	Date (Month/day)__________	Time (24-hour)______

c) Documented Patient Challenges

	Not reported in 4b	'Yes' in 4b & none exist	'Yes' in 4b & challenges exist	Summary of Challenges
Medical	☐	☐	☐	__________________________
Functional	☐	☐	☐	__________________________
Cognitive	☐	☐	☐	__________________________
Mental	☐	☐	☐	__________________________
Social Support	☐	☐	☐	__________________________
Prescription drug	☐	☐	☐	__________________________
Other (state)	☐	☐	☐	__________________________

d) Evidence that a Rehab Plan was Created

	Medical	Functional	Cognitive	Mental	Soc. Support	Drug	Other
i) Not reported/unclear if patient has challenges; No plans created.	☐	☐	☐	☐	☐	☐	☐
ii) Documented that patient has no challenges in this area.	☐	☐	☐	☐	☐	☐	☐
iii) Patient has documented challenges in this area, & no rehab plan created.	☐	☐	☐	☐	☐	☐	☐
iv) Patient has documented challenges in this area, & rehab plan created. If so, summarize the rehab plans.	☐	☐	☐	☐	☐	☐	☐

Medical ____

____

Functional ____

____

Cognitive ____

____

Mental ____

____

Soc Support ____

____

Prescription drug ____

____

Other (state) ____

____

5. **Evidence That Rehab Plans Were Enacted During Patient Stay**

	Medical	Functional	Cognitive	Mental	Soc Support	Drug	Other
i) Patient challenges not clearly documented or absent, & rehab plans not enacted.	☐	☐	☐	☐	☐	☐	☐
ii) Clearly documented that patient has no challenges in this area; no rehab plans enacted.	☐	☐	☐	☐	☐	☐	☐
iii) While patient has clearly documented challenges in this area, no evidence that rehab was enacted.	☐	☐	☐	☐	☐	☐	☐
iv) Provider enacted rehab but patient refused or was unable to participate. If so, include all dates (Month/DD)	☐	☐	☐	☐	☐	☐	☐
v) Provider enacted rehab plan. If so, include all dates (Month/DD)	☐	☐	☐	☐	☐	☐	☐

6. **Patient Progress Reported**

	Medical	Functional	Cognitive	Mental	Soc Support	Drug	Other
i) Patient challenges not clearly documented or absent; no patient progress reported	☐	☐	☐	☐	☐	☐	☐
ii) Clearly documented that patient has no challenges in this area; no patient progress reported	☐	☐	☐	☐	☐	☐	☐
iii) While patient has clearly documented challenges in this area; no patient progress reported.	☐	☐	☐	☐	☐	☐	☐

iv) Patient has clearly documented challenges in this area; progress is reported. If so, include all dates (Month/DD)	☐ ____ ____ ____ ____ ____	☐ ____ ____ ____ ____ ____	☐ ____ ____ ____ ____ ____	☐ ____ ____ ____ ____ ____	☐ ____ ____ ____ ____ ____	☐	☐ ____ ____ ____ ____ ____

7. Description of Patient Profile at the end of SAC care

	Not Provided	Provided	Summary of Description
Medical	☐	☐	____________________
Functional	☐	☐	____________________
Cognitive	☐	☐	____________________
Mental	☐	☐	____________________
Soc Support	☐	☐	____________________
Prescription drug	☐	☐	____________________
Other (state)	☐	☐	____________________

8. Evidence that a hospital-to-home transition plan has been initiated

	No Rehab Needed	No plan Provided	Plan Provided	Summary of Plan
Medical	☐	☐	☐	____________________
Functional	☐	☐	☐	____________________
Cognitive	☐	☐	☐	____________________
Mental	☐	☐	☐	____________________
Soc Support	☐	☐	☐	____________________
Prescription drug	☐	☐	☐	____________________
Other (state)	☐	☐	☐	____________________
